# Is the combination of enalapril and losartan irrational?

**DOI:** 10.4103/0253-7613.41047

**Published:** 2008

**Authors:** D.M. Parmar, S.P. Jadav

**Affiliations:** Department of Pharmacology, M.P. Shah Medical College, Jamnagar - 361 008, Gujarat, India. E-mail: drdinesh06@rediffmail.com

Different views have been expressed on the topic of ‘combination therapy of angiotensin converting enzyme (ACE) inhibitors and angiotensin receptor blockers (ARB): rational or irrational?’ in the form of correspondences published in the Indian Journal of Pharmacology.[1–4]

The discussion on this topic started with the editorial by Gautam and Aditya in which they had suggested that the combination of enalapril and losartan is irrational.[5] Tandon,[1] Sharma *et al*.,[2] and Petroianu[4] have argued that the combination of ACE inhibitors and ARB is rational based on evidences obtained from clinical trials. Gautam and Aditya[3] categorized this combination as controversial rather than rational, also citing the results of related clinical trials; they had also opined that the long-awaited results of the ONTARGET (Ongoing Telmisartan Alone and in combination with Ramipril Global Endpoint Trial) may help to define clearly the status of such a combination.

The results of the ONTARGET study have been published in a recent issue of the New England Journal of Medicine; the study found that the combination of telmisartan (an ARB) and ramipril (an ACE inhibitor) was associated with more adverse events, without offering any increase in benefits. This particular combination showed higher rates of hypotensive symptoms, syncope, renal dysfunction, and hyperkalemia, with a trend toward an increased risk of renal dysfunction requiring dialysis.[6] The VALIANT (Valsartan in Acute Myocardial Infarction Trial) study showed additional adverse effects, including hypotension and renal dysfunction, with a combination of an ARB and an ACE inhibitor.[7] Both the VALIANT and the ONTARGET trials added an ARB to an evidence-based dose of an evidence-based ACE inhibitor.[8] On the other hand, two heart failure studies, namely the Valsartan Heart Failure Trial (Val-HeFT) and the Candesartan in Heart Failure: Assessment of Reduction in Mortality and Morbidity-Added (CHARM-Added), showed that additional benefits were to be had by combining an ARB with an ACE inhibitor.[9,10] However, in both these studies (VAL-HeFT and CHARM-Added), an ARB was combined with the physicians' choice of the type and regimen of ACE inhibitor and, therefore, whether the benefits of combination therapy observed in these studies were due to the condition studied (heart failure) or the type or regimen of the ACE inhibitor used is uncertain.[8] A meta-analysis of randomized controlled trials showed that combination therapy with an ARB plus an ACE inhibitor in subjects with symptomatic left ventricular dysfunction was accompanied by marked increases in adverse effects.[11]

To sum up, available evidence-based information indicates that the combination of an ARB and an ACE inhibitor is irrational.
